# Supplementary material for: SyNDI: synchronous network data integration framework
Source: BMC Bioinformatics. 2018 Nov 6;19:403. doi: 10.1186/s12859-018-2426-5 (PMC6219086; doi:10.1186/s12859-018-2426-5)
Supplement: Supplementary file 5 — Files of the example presented in Additional file 4. This additional file is a zip package that contains all files used in Additional file 4. Detailed descriptions of each of these files are presented in a README file included in this zip package. (GZ 30411 kb) [file 12859_2018_2426_MOESM5_ESM.gz › SyNDITools/Meme2Fimo/meme_4.9.0_4.tar.gz!/meme_4.9.0/website/html/mast-output.html]

MAST - Output


|  |  |  |  |  |
| --- | --- | --- | --- | --- |
|  | MEME Suite The MEME Suite web application requires the use of JavaScript Javascript doesn't seem to be available on your browser. MAST -- Motif Alignment and Search Tool *Motif search tool* --- Search Results The MAST results consist of - The **inputs** to MAST including: 1. The **sequence databases** showing the sequence and residue counts. [View] 2. The **motifs** showing the name, width, best scoring match and similarity to other motifs. [View] - The **search results** showing top scoring sequences with tiling of all of the motifs matches shown for each of the sequences. [View] - The **program** details including: 1. The **version** of MAST and the date it was released. [View] 2. The **reference** to cite if you use MAST in your research. [View] 3. The **command line summary** detailing the parameters with which you ran MAST. [View] - An **explanation** of how to interpret MAST results. [View] Match Scores The match score of a motif to a position in a sequence is the sum of the score from each column of the position-dependent scoring matrix corresponding to the letter at that position in the sequence. For example, if the sequence is ``` TAATGTTGGTGCTGGTTTTTGTGGCATCGGGCGAGAATAGCGC ======== ``` and the motif is represented by the position-dependent scoring matrix (where each row of the matrix corresponds to a position in the motif) Position \| A | C | G | T |
| 1 | 1.447 | 0.188 | -4.025 | -4.095 |
| 2 | 0.739 | 1.339 | -3.945 | -2.325 |
| 3 | 1.764 | -3.562 | -4.197 | -3.895 |
| 4 | 1.574 | -3.784 | -1.594 | -1.994 |
| 5 | 1.602 | -3.935 | -4.054 | -1.370 |
| 6 | 0.797 | -3.647 | -0.814 | 0.215 |
| 7 | -1.280 | 1.873 | -0.607 | -1.993 |
| 8 | -3.076 | 1.035 | 1.414 | -3.913 |

then the match score of the fourth position in the sequence (underlined)
would be found by summing the score for T in position 1, G in position 2 and
so on until G in position 8. So the match score would be

```
               score = -4.095 + -3.945 + -3.895 + -1.994
                       + -4.054 + -0.814 + -1.933 + 1.414 
                     = -19.316
```

The match scores for other positions in the sequence are calculated in the
same way. Match scores are only calculated if the match completely fits within
the sequence. Match scores are not calculated if the motif would overhang
either end of the sequence. **Note:** Scores for any
IUPAC ambiguous characters appearing
in a sequence are calculated as the weighted average of the scores
of letters that match the ambiguous character. The weights are the
background frequencies of the letters specified to MAST.

### P-values

MAST reports all matches of a sequence to a motif or group of
motifs in terms of the p-value of the match. MAST considers the
p-values of four types of events:

- **position p-value:** the match of a
  single position within a sequence to a given motif,
- **sequence p-value:** the best match
  of any position within a sequence to a given motif,
- **combined p-value:** the combined
  best matches of a sequence to a group of motifs, and
- **E-value:** observing a combined
  p-value at least as small in a random database of the same
  size.

All p-values are based on a random sequence model that assumes each
position in a random sequence is generated according to the average
letter frequencies of all sequences in the database being searched.

#### Position p-value

The p-value of a match of a **given position** within a
sequence to a **motif** is defined as the probability of a
randomly selected position in a randomly generated sequence having
a **match score** at least as large as that
of the given position.
**Note:**If MAST is combining reverse complement DNA strands,
the position p-value is **not** corrected for multiple tests.

#### Sequence p-value

The p-value of a match of a **sequence** to a **motif**
is defined as the probability of a randomly generated sequence of
the same length having a match score at least as large as the
largest match score of any position in the sequence.

#### Combined p-value

The p-value of a match of a **sequence** to a **group of
motifs** is defined as the probability of a randomly generated
sequence of the same length having sequence p-values whose
**product** is at least as small as the product of the sequence
p-values of the matches of the motifs to the given sequence.

#### E-value

The E-value of the match of a **sequence** in a
**database** to a a **group of motifs** is defined as the
expected number of sequences in a random database of the same size
that would match the motifs as well as the sequence does and is
equal to the combined p-value of the sequence times the number of
sequences in the database.

### Input Database and Motifs

This section shows information on the database that was searched
and the motifs in the search query. The database section
[View]
gives the date the database was last updated as well as the number of
sequences and total sequence characters in it. The motifs 
[View]
are listed by motif number. The width and subsequence which would be
given the best possible score for each motif is shown. If there is
more than one motif in the query, all pairwise correlations between
the motifs are shown. The correlations can range from -1 to +1,
with +1 meaning that the shorter motif is exactly identical to part
or all of the longer motif. High correlations can cause some
combined p-values and E-values to be inaccurate (too low). It may
be advisable to remove enough motifs from the query to insure that
no pairs of motifs have high correlations. Any high correlations
are indicated along with the suggestion that one of the motifs be
removed from the query.

### Top Scoring Sequences

MAST lists the names, *E*-values, link to
expanded results
and motif block diagram of all
sequences whose E-value is less than
the set threshold. Sequences shorter than one or more of the motifs are
skipped. The sequences are sorted by increasing E-value. The *E*-value
threshold is set to 10 for the web server by default.

#### Motif Block Diagrams

Motif block diagrams show the order and spacing of non-overlapping
matches to the motifs in each high-scoring sequence. Motif
occurrences are determined based on the position p-value of matches to the motif. For each
high scoring sequence the in the output, diagrams are shown like this:

|  |  |
| --- | --- |
|  | Motif 1 |

|  |  |
| --- | --- |
|  | Motif 2 |

|  |  |
| --- | --- |
|  | Motif 3 |

+

-

0

100

200

300

400

500

The vertically central line shows the extent of the sequence relative to
the lengths of the other sequences in the database. The ruler at the bottom
gives an indication of the actual length of the sequence. The coloured
blocks are motif occurrences. Blocks above the line are on the given strand,
blocks below the line are on the reverse complement strand (DNA only). The
colour and border of the block can be used to identify the motif by using
the legend. The height of the block can be used to get an indication of the
significance of the match with taller blocks being more significant. If you
place the mouse cursor over any of the motif blocks then the "title" text
will be displayed. It lists the name of the motif, the *p*-value of the
occurrence, the frame (translated DNA only) and the extent.

### Expanded Results

By clicking a link next to the *E*-value the expanded results become visible.
The expanded results include the sequence comment, combined *p*-value,
the best frame (for translated sequences) and the annotated sequence.

#### Annotated Sequences

In the expanded results view, MAST shows an annotated sequence by printing the sequence
along with the position and strength of all the non-overlapping motif occurrences for a user
selectable portion of the sequence. Dragging the buttons below the motif block diagram
modifies the visible portion of the annotated sequence. The four lines above each motif occurrence
contain, respectively,

- the motif name of the occurrence,
- the position p-value of the
  occurrence,
- the best possible match to the motif, and
- a plus sign (`+') above each letter in the occurrence that has
  a positive match score to the motif.

The **best possible match** to a motif is the sequence of
letters which would achieve the highest match score.

When peptide motifs are used to search nucleotide sequences, the
reading frame (a, b or c) of each match is indicated with the motif
name and the peptide translation of the matching sequence is
shown just above the motif occurrence.

### Sample MAST Search Results

Here is an actual MAST search results file
of a search of a peptide database with peptide motifs.
